# The Impact of Sorafenib in Combination with Transarterial Chemoembolization on the Outcomes of Intermediate-Stage Hepatocellular Carcinoma

**DOI:** 10.31557/APJCP.2021.22.4.1217

**Published:** 2021-04

**Authors:** Masaki Kaibori, Hideyuki Matsushima, Morihiko Ishizaki, Hisashi Kosaka, Kosuke Matsui, Shuji Kariya, Kengo Yoshii, Mitsugu Sekimoto

**Affiliations:** 1 *Department of Surgery, Kansai Medical University, Hirakata, Osaka, Japan. *; 2 *Department of Radiology, Kansai Medical University, Hirakata, Osaka, Japan. *; 3 *Department of Mathematics and Statistics in Medical Sciences, Kyoto Prefectural University of Medicine, Kyoto, Japan. *

**Keywords:** Sorafenib, transarterial chemoembolization, refractory, intermediate, stage hepatocellular carcinoma

## Abstract

**Background:**

We investigated the treatment outcomes and hepatic reserve of transarterial chemoembolization (TACE)-refractory patients with recurrent advanced hepatocellular carcinoma (HCC) treated with TACE plus sorafenib.

**Methods:**

Forty-one patients with intermediate-stage HCC defined as being TACE refractory on imaging were treated with sorafenib and TACE between 2009 and 2012 and comprised the combination treatment group. Twenty-nine patients who received repeated TACE after becoming refractory to TACE between 2005 and 2008 comprised the TACE continuation group.

**Results:**

Although the interval between successive rounds of TACE was significantly shorter before the patients developed TACE refractoriness, it was significantly longer after the development of TACE refractoriness, in the combination treatment group compared with the TACE continuation group. The appearance of extrahepatic spread and/or vascular invasion differed significantly between the two groups. The median overall survival was significantly longer in the combination treatment group than in the TACE continuation group (20.5 vs. 15.4 months, respectively; hazard ratio = 2.04; 95% confidence interval = 1.20–3.48). The 3-year overall survival rate was 33.4% in the combination treatment group and 3.5% in the TACE continuation group. Downstaging of the Child–Pugh class was significantly less frequent in the combination treatment group than in the TACE continuation group. In COX proportional hazards analyses, sorafenib plus TACE resulted in a better prognosis compared with repeated TACE.

**Conclusions:**

Treatment with sorafenib plus TACE in TACE-refractory patients with intermediate-stage HCC resulted in longer intervals between TACE rounds, better maintenance of hepatic reserve, and significantly longer OS compared with repeated TACE.

## Introduction

Hepatocellular carcinoma (HCC) is the fifth and eighth most common malignancy in men and women, respectively, and more than 500,000 new cases are diagnosed worldwide each year (Bosch et al., 1999; Bosch et al., 2004; Arzumanyan et al., 2013). Transarterial chemoembolization (TACE) confers a survival benefit in patients with intermediate-stage HCC (Llovet et al., 2003). Most clinical practice guidelines, including those of the Barcelona Clinic Liver Cancer (BCLC) (Forner et al., 2018), European Association for the Study of the Liver (European Association for the Study of the Liver, 2018), American Association for the Study of Liver Diseases (AASLD) (Heimbach et al., 2018; Bruix et al., 2011), Asian Pacific Association for the Study of the Liver (APASL) (Omata et al., 2017), and Japan Society of Hepatology (JSH) (Kudo et al., 2016), recommend the use of TACE for these patients, and TACE has emerged as the standard of care. Because of the high tumor recurrence rate after TACE, this procedure is usually repeated multiple times. However, repetition of TACE may lead to deteriorated liver function, which results in a poor patient prognosis (Hiraoka et al., 2017). 

Sorafenib is an oral multikinase inhibitor that targets RAF, platelet-derived growth factor receptor, and vascular endothelial growth factor (VEGF) receptor, among other kinases, thereby exerting both antiangiogenic and antitumor effects (Wilhelm et al., 2008). We speculate that sorafenib suppresses the surge in proangiogenic factors that occurs after TACE. It was shown that sorafenib significantly prolongs progression-free survival (PFS) and overall survival (OS) in patients with advanced HCC (Llovet et al., 2008; Cheng et al., 2009). Sorafenib has become the standard treatment for patients with advanced unresectable HCC. Although many clinical trials (e.g., Post-TACE, SPACE, and TACE-2) have evaluated the combination of sorafenib and TACE, no significant benefits of this combination treatment have been reported (Kudo et al., 2011; Lencioni et al., 2016; Meyer et al., 2017). Meanwhile, the TACTICS phase II study, which investigated patients with BCLC-A and BCLC-B lesions in Japan, yielded epoch-making results (Kudo et al., 2020). A significantly longer median PFS was observed in the TACE plus sorafenib combination group compared with the TACE only group; the authors are awaiting the OS results. Combination therapy with TACE and sorafenib is expected to improve the prognosis of patients with HCC.

In this study, we investigated the treatment outcomes and hepatic reserve in TACE-refractory patients with recurrent advanced HCC after treatment with TACE plus sorafenib.

## Materials and Methods

*Patients*

Of 94 patients with recurrent HCC after resection who received sorafenib between 2009 and 2012, 41 with intermediate-stage HCC defined as being refractory to TACE by imaging were treated with sorafenib and TACE (combination treatment group). Of 67 patients receiving TACE for postoperative recurrence in our department between 2005 and 2008, 29 defined as being refractory to TACE and who received repeated TACE served as the control group (TACE continuation group). The definition of TACE refractoriness was based on the criteria of the JSH–Liver Cancer Study Group of Japan, a revised version of which was reported in 2014 (Kudo et al., 2014). All 70 patients enrolled in this retrospective cohort study had Child–Pugh class A liver function and an Eastern Cooperative Oncology Group performance status of 0–1.

*Methods*

As the primary endpoint, OS was analyzed retrospectively in both groups. Hepatic reserve was analyzed after patients became refractory to TACE. The Child–Pugh score was compared between the groups every 6 months after the development of TACE refractoriness, up to 36 months. The survey ended in December 2015 for the combination treatment group and in December 2011 for the TACE continuation group, after an observation period of 3 years in both groups.

*TACE technique*

The right femoral artery was assessed by the Seldinger technique using an 18-gauge needle, and a 5-Fr sheath was then inserted. In all patients, HCC and vascularization were evaluated by celiac arteriography, computed tomography (CT) during hepatic arteriography, and CT during arterioportography. The arteries feeding the HCC were selectively catheterized using a coaxial catheter system with a 5- or 4-Fr catheter and microcatheter as distally as possible according to tumor size and location. In cases of multiple HCCs, TACE was performed from the segmental, subsegmental, lobar, and/or proper arteries. Epirubicin 20–50 mg (Epirubicin^®^; Nippon Kayaku, Tokyo, Japan) emulsified in 1–8 mL iodized oil (Lipiodol^®^ Ultra-Fluid; Guerbet, Paris, France), 60–120 mg miriplatin (Miripla^®^; Sumitomo Dainippon Pharma, Osaka, Japan), or 50–100 mg cisplatin (IA-call^®^; Nippon Kayaku) were used as the anti-cancer drugs depending on the total tumor volume, embolized area, and hepatic function. Gelatin sponge particles (Gelpart^®^; Nippon Kayaku or Gelfoam^®^; Upjohn, Kalamazoo, MI, USA) were used as the embolic material. Miriplatin became available in 2010 in Japan, whereas the other drugs were available throughout the study period. There were no changes in the TACE procedure during the study period. Neither drug-eluting bead TACE nor balloon-occluded TACE was used in this study.

*Definition of TACE refractoriness*

The definition of TACE refractoriness was based on the 2014 JSH consensus guidelines, and we judged a patient as being TACE refractory when any of the following three conditions were met (Kudo et al., 2014). (1) The treated tumor exhibited two or more consecutive insufficient responses (viable lesion > 50%) and two or more consecutive increases in tumor number compared with before the previous TACE round, even after changing the chemotherapeutic agent and/or reanalysis of the feeding artery by response evaluation CT/magnetic resonance imaging (MRI) 1–3 months after adequate selective TACE. (2) Vascular invasion and/or extrahepatic metastasis appeared. (3) There were continuous elevations in tumor marker concentrations immediately after TACE despite slight transient decreases.

*Treatment strategy using sorafenib*

In the combination treatment group, sorafenib was administered orally at 400 or 200 mg once daily. Dose reduction or interruption of sorafenib therapy was allowed depending on the type and severity of the adverse events. Concerning the criteria for TACE combined with sorafenib, repeated TACE was performed for clinically determined cases in which sorafenib therapy could not be continued because of adverse effects and/or in which the HCC improved with combination therapy or, alternatively, additional TACE was possible because of adequate liver function even if the cancer had progressed. Repeated TACE was also performed when a new intrahepatic lesion appeared, or another lesion progressed even if the target lesion was stable or improved. Sorafenib treatment was continued without interruption except during the first 7 days after TACE. Evaluation of the response was performed using CT or MRI at 1–3 months after TACE in both groups.

*Statistical analysis*

We compared the patients’ clinical characteristics between the groups using Wilcoxon’s rank-sum test, the chi-squared test, or Fisher’s exact test. The probabilities of time to extrahepatic spread, advanced stage, progression from Child–Pugh class A to B or C, and OS according to treatment type were calculated using the Kaplan–Meier method. Hazard ratios (HRs) for survival and their 95% confidence intervals (CIs) were estimated using a univariate Cox model. Multivariate analysis was performed using a Cox proportional hazards model. The following variables were examined as potential prognostic predictors: age, maximum tumor size, Child–Pugh score, concentrations of α-fetoprotein and protein induced by vitamin K absence II (PIVKA-II), post-treatment, and TACE. A two-sided p value < 0.05 was considered statistically significant. All statistical analyses were performed using R version 3.4.3 (R Foundation for Statistical Computing, Vienna, Austria) with the survival packages.

## Results

Table 1 summarizes the clinical characteristics of both groups after the patients developed TACE-refractory disease, and Table 2 summarizes the clinical characteristics after each study treatment. No differences were detected between the groups in terms of sex, age, number of tumors, tumor location, serum level of α-fetoprotein or PIVKA-II , hepatitis B surface antigen level, hepatitis C virus antibody positivity, Child–Pugh score, BCLC stage, the time from the first round of TACE to developing TACE refractoriness, number of sessions of TACE before developing TACE refractoriness, additional rounds of TACE after developing TACE refractoriness, or the effect of the first session of TACE after developing TACE refractoriness. The maximum tumor size was significantly greater in the combination treatment group than in the TACE continuation group. Although the median interval between successive rounds of TACE was not significantly different between the combination treatment group and TACE continuation group before the development of TACE refractoriness (4.1 vs. 6.8 months; p = 0.043), it was significantly longer in the combination treatment group than in the TACE continuation group after the development of TACE refractoriness (5.0 vs. 3.3 months; p = 0.002). Post-treatment was performed in 17 patients (41%) in the combination treatment group, compared with 4 patients (14%) in the TACE continuation group (p = 0.026). The rates of extrahepatic lesions and vascular invasion differed significantly between the two groups. 

**Figure 1.**
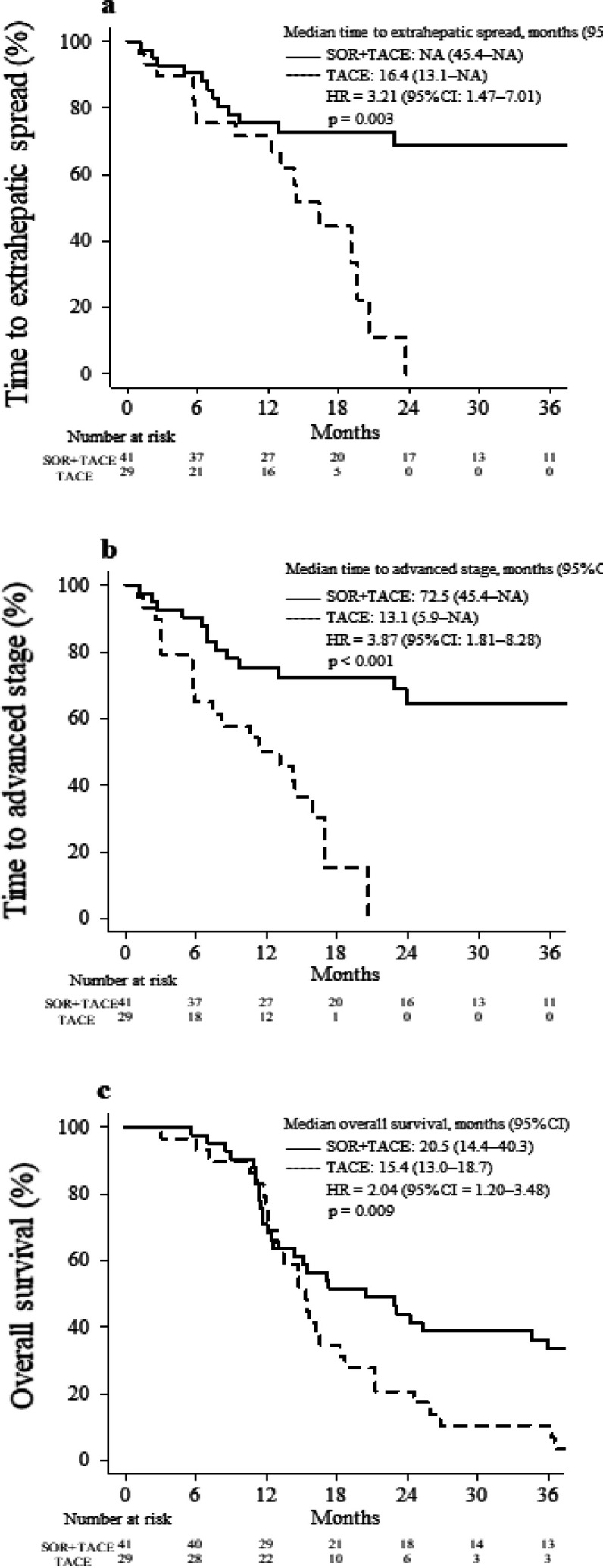
Survival Outcomes in the Combination Treatment and TACE Continuation Groups. (a), Time to extrahepatic spread; (b), Time to progression to advanced-stage disease; (c), Overall survival rate. CI, confidence interval; HR, hazard ratio; SOR, sorafenib; TACE, transarterial chemoembolization

Figure 1 presents a comparison of the long-term outcomes between the two groups. The median follow-up period was 20.5 months in the combination treatment group and 15.4 months in the TACE continuation group. The median time to extrahepatic spread was significantly longer in the combination treatment group than in the TACE continuation group (not reached vs. 16.4 months; HR = 3.21; 95% CI = 1.47–7.01; p= 0.003; Figure 1a). The proportion of patients without extrahepatic spread within 2 years was 68.7% in the combination treatment group and 0.0% in the TACE continuation group. The median time to progression (TTP) to advanced stage was significantly longer in the combination treatment group than in the TACE continuation group (72.5 vs. 13.1 months; HR = 3.87; 95% CI = 1.81–8.28; p < 0.001; Figure 1b). The proportion of patients without progression to an advanced stage within 1 year was 75.4% in the combination treatment group and 50.2% in the TACE continuation group. The median OS was significantly longer in the combination treatment group than in the TACE continuation group (20.5 vs. 15.4 months, respectively; HR = 2.04; 95% CI = 1.20–3.48; p = 0.009; Figure 1c). The 3-year OS rates were 33.4% and 3.5% in the combination treatment and TACE continuation groups, respectively. 

**Figure 2:**
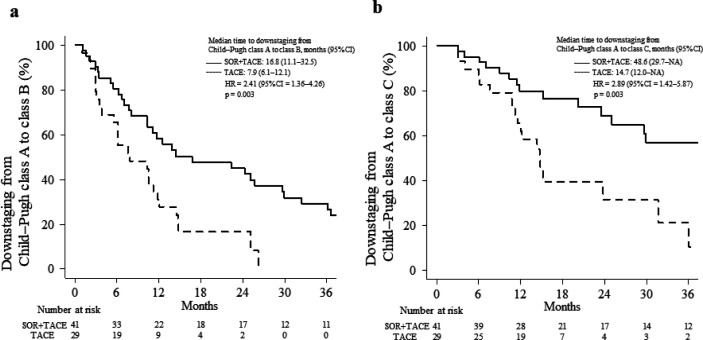
Downstaging of the Child–Pugh Class in the Combination Treatment and TACE Continuation Groups. (a) Downstaging from Child–Pugh class A to B. (b) Downstaging from Child–Pugh class A to C. CI, confidence interval; HR, hazard ratio; SOR, sorafenib; TACE, transarterial chemoembolization; NA, not applicable

Figure 2 shows a comparison of the deterioration in liver function between the two groups. The median time to downstaging from Child–Pugh class A to B was significantly longer in the combination treatment group than in the TACE continuation group (16.8 vs. 7.9 months, respectively; HR = 2.41; 95% CI = 1.36–4.26; p = 0.003; Figure 2a). The proportion of patients in whom no downstaging from Child–Pugh class A to B occurred within 2 years was 42.4% in the combination treatment group and 16.6% in the TACE continuation group. The median time to downstaging from Child–Pugh class A to C was significantly longer in the combination treatment group than in the TACE continuation group (48.6 vs. 14.7 months, respectively; HR = 2.89; 95% CI = 1.42–5.87; p = 0.003; Figure 2b). The proportion of patients in whom no downstaging from Child–Pugh class A to C occurred within 3 years was 56.8% in the combination treatment group and 10.5% in the TACE continuation group.

Table 3 presents the results from a multivariate Cox proportional hazards model analyzing several covariates as prognostic factors for OS. TACE-only treatment was identified as the only independent predictor of OS (HR = 2.08; 95% CI = 0.48–1.08; p = 0.028).

**Table 1:** Comparison of Clinical Characteristics between the Combination Treatment and *TACE* Continuation Groups

Characteristic	SOR+TACE (n = 41)	TACE (n = 29)	p
Sex			0.856
Male	32 (78%)	24 (83%)	
Female	9 (22%)	5 (17%)	
Age, years	70 (57–81)	71 (57–76)	0.358
Number of tumors			0.664
<4	16 (39%)	9 (31%)	
≥4	25 (61%)	20 (69%)	
Tumor size, mm	21 (7–81)	14 (6–33)	0.049
Tumor location			0.426
Hemiliver	12 (29%)	12 (41%)	
Whole liver	29 (71%)	17 (59%)	
Alpha-fetoprotein, ng/mL	11 (2–769)	26 (3–756)	0.208
PIVKA-II, mAU/mL	32 (12–5908)	48 (20–1609)	0.194
HBsAg			1
Negative	34 (83%)	25 (86%)	
Positive	7 (17%)	4 (14%)	
HCVAb			0.401
Negative	18 (44%)	9 (31%)	
Positive	23 (56%)	20 (69%)	
Child–Pugh score			0.545
5	24 (59%)	14 (48%)	
6	17 (41%)	15 (52%)	
BCLC stage B			1
No	0 (0%)	0 (0%)	
Yes	41 (100%)	29 (100%)	
Period from first TACE session to refractory, months	10.9 (0.8–64)	18.2 (4.7–63)	0.147
Number of TACE sessions before TACE-refractory	3 (2–7)	3 (2–4)	0.435
Interval between TACE sessions before TACE-refractory, months	4.1 (0.3–19.3)	6.8 (2.3–18.2)	0.043

Data are shown as n (%) or median (90% confidence interval). p-values in bold font indicate; *PIVKA-II* protein induced by vitamin K absence-II, *HCC* hepatocellular carcinoma, *HBsAg* hepatitis B surface antigen, *HCVAb* hepatitis C virus antibody, *TACE *transarterial chemoembolization

**Table 2:** Clinical Characteristics and Response Rates after Each Study Treatment

Characteristic	SOR+TACE (n = 41)	TACE (n = 29)	P
Initial dose of sorafenib			
200 mg/400 mg	21 (51%)/20 (49%)		
Duration of sorafenib treatment, months	11.6 (3.7–52.3)		
Interval between TACE sessions after TACE-refractory, months	5.0 (2.0–11.0)	3.3 (1.4–7.6)	0.002
Number of additional TACE sessions after TACE-refractory	2 (1–5)	2 (1–6)	0.466
Effect of first TACE after TACE-refractory			0.296
CR	0 (0%)	0 (0%)	
PR	2 (5%)	3 (10%)	
SD	30 (73%)	16 (55%)	
PD	9 (22%)	10 (34%)	
Post-treatment			0.026
No	24 (59%)	25 (86%)	
Yes	17 (41%)	4 (14%)	
Extrahepatic spread and/or vascular invasion			0.026
Negative	25 (61%)	9 (31%)	
Positive	16 (39%)	20 (69%)	

PIVKA-II, protein induced by vitamin K absence-II; TACE*,* transarterial chemoembolization; OS, overall survival; HR, hazard ratio; CI, confidence interval

**Table 3:** Cox Proportional Hazards Regression Analysis of Overall Survival in TACE-refractory Patients with Advanced Hepatocellular Carcinoma

Variable	OS		
	HR	(95% CI)	p
Age ≥70 years (vs. <70 years)	0.56	(1.79–0.31)	0.051
Tumor size ≥30 mm (vs. <30 mm)	1.77	(0.57–0.84)	0.134
Child–Pugh score 6–7 (vs. 5)	1.78	(0.56–0.94)	0.076
Alpha-fetoprotein ≥200 ng/mL (vs. <200 ng/mL)	2.05	(0.49–0.94)	0.071
PIVKA-II ≥1000 mAU/mL (vs. <1000 mAU/mL)	1.26	(0.79–0.46)	0.654
No post-treatment (vs. yes)	1.41	(0.71–0.70)	0.342
TACE (vs. sorafenib+TACE)	2.08	(0.48–1.08)	0.028

PIVKA-II, protein induced by vitamin K absence-II; TACE, transarterial chemoembolization; OS, overall survival; HR, hazard ratio; CI, confidence interval

**Table 4:** Trials of Sorafenib Alone and in Combination with TACE in TACE-refractory Patients with Advanced HCC

Trial/Authors	BCLC stage	Design	OS	TTP/PFS	Characteristics of each test/Differences from the current study
Post-TACE [Kudo et al., 2011]	Intermediate	Sorafenib vs. placebo	29.7 months vs. NE; *p* = 0.790; HR = 1.06 (95% CI = 0.69–1.64)	5.4 months vs. 3.7 months; *p* = 0.252; HR = 0.87 (95% CI = 0.70–1.09)	Number of prior TACE sessions: 1–2 Median time from last TACE to randomization: 1.4–3.3 months Median period of sorafenib administration: 4.3 months
SPACE [Lencioni et al., 2016]	Intermediate	DEB-TACE+sorafenib vs. DEB-TACE	NR vs. NR; *p* = 0.295; HR = 0.898 (95% CI = 0.606–1.33)	5.6 months vs. 5.5 months; *p* = 0.072; HR = 0.797 (95% CI = 0.588–1.08)	Scheduled TACE (1, 3, 7 months, every 6 months thereafter) Median period of sorafenib administration: 5.3 months
TACE-2 [Meyer et al., 2017]	Intermediate	DEB-TACE+sorafenib vs. DEB-TACE	21.0 months vs. 19.9 months; *p* = 0.57; HR = 0.91 (95% CI = 0.67–1.24)	7.9 months vs. 7.8 months; *p* = 0.94; HR = 0.99 (95% CI = 0.77–1.27)	Additional TACE was allowed even before PD according to the investigator’s judgment Median period of sorafenib administration: 4.3 months
TACTICS [Kudo et al., 2020]	Early/intermediate/advanced	TACE+sorafenib vs. TACE	Not analyzed as of April 28, 2020	25.2 months vs. 13.5 months; *p* = 0.006; HR = 0.59 (95% CI = 0.41–0.87)	Number of prior TACE sessions: 0–2 Rate of early-stage cancer: 33.8% vs. 43.4% Median period of sorafenib administration: 9.7 months
STAB [Sato et al., 2018]	Advanced	TACE+sorafenib	17.3 months	5.4 months	Presence of clear margin and intrahepatic tumors affected prognosis BCLC-C (macrovascular invasion or extrahepatic spread)
Ogasawara [Ogasawara et al., 2014]	Intermediate	Sorafenib vs. TACE	25.4 months vs. 11.5 months; *p* = 0.003	22.3 months vs. 7.7 months; *p*=0.001	Benefits of sorafenib therapy in TACE-refractory patients
Arizumi [Arizumi et al., 2015]	Intermediate	Sorafenib vs. TACE	24.7 months vs. 13.6 months; *p* = 0.002	No data	Benefits of sorafenib therapy in TACE-refractory patients
Ohki [Ohki et al., 2015]	Intermediate	Sorafenib vs. TACE	28.7 months vs. 15.6 months; *p* < 0.01	6.3 months vs. 3.5 months; *p* < 0.01	Benefits of sorafenib therapy in TACE-refractory patients
Our study	Intermediate	TACE vs. TACE+sorafenib	15.4 months vs. 20.5 months; *p* = 0.009; HR = 2.04 (95% CI = 1.20–3.48)	4.9 months vs. 6.5 months; *p* = 0.399; HR = 1.24 (95% CI = 0.75–2.04)	Benefits of combined sorafenib treatment and TACE in TACE-refractory patients Number of prior rounds of TACE (median): 3 Median period of sorafenib administration: 11.6 months

BCLC, Barcelona Clinic Liver Cancer; OS, overall survival; TTP, time to progression; PFS, progression-free survival; TACE, transarterial chemoembolization; NE, not estimable owing to immaturity of the data; HR, hazard ratio; CI, confidence interva; NR, not reached; DEB-TACE, drug-eluting beads TACE; PD, progressive disease

## Discussion

In clinical guidelines such as those proposed by the BCLC, AASLD, APASL, and JSH, TACE is recommended as a standard treatment for intermediate-stage HCC (Bruix et al., 2011; Kudo et al., 2016; Omata et al., 2017; European Association for the Study of the Liver, 2018; Forner et al., 2018; Heimbach et al., 2018). In clinical practice, repeated TACE may be beneficial, but repeated TACE after patients become refractory to TACE may result in inadequate treatment and deterioration of the hepatic reserve (Raoul et al., 2014). Although molecular targeted drugs such as sorafenib and lenvatinib are recommended for patients with TACE-refractory HCC (Kudo et al., 2014), TACE is often continued in patients with intrahepatic HCC even after developing TACE refractoriness. Although the number of anticancer agents that can be used to treat advanced HCC has increased in recent years, and sequential therapy using multiple anticancer agents has been examined, it is most important to prevent deterioration of hepatic reserve.

In this single-center, retrospective, non-randomized trial, we compared OS and hepatic reserve between the combination treatment and TACE continuation groups among patients who met the definition of TACE refractoriness. The median OS was significantly longer in the combination treatment group than in the TACE continuation group (20.5 vs. 15.4 months; HR = 2.04; 95% CI = 1.20–3.48; p = 0.009; Figure 1c), and the time to Child–Pugh class downstaging was also significantly longer in the combination treatment group (Figure 2). In the Cox proportional hazards analysis, sorafenib plus TACE was associated with a better prognosis compared with repeated TACE (Table 3).

In addition, the time to appearance of extrahepatic spread and the TTP to advanced stage were significantly prolonged in the combination treatment group than in the TACE continuation group. Compared with the TACE continuation group, the interval between successive rounds of TACE in the combination treatment group was significantly shorter before the patients became TACE refractory but significantly longer after the patients became refractory. Combination therapy with sorafenib plus TACE increased the interval until the next TACE session, enhanced maintenance of the hepatic reserve, and improved the transition to post-treatment. These reasons may explain why OS was significantly better in the combination treatment group than in the TACE continuation group.

This study differs from previous TACE combination trials and sorafenib studies conducted in patients with TACE-refractory advanced HCC (Table 4). First, regarding differences in the study population and design, previous trials of TACE combination therapy (Post-TACE, SPACE, TACE-2, and TACTICS) (Kudo et al., 2011; Lencioni et al., 2016; Meyer et al., 2017; Kudo et al., 2020) targeted mainly patients with early- or intermediate-stage disease, and the number of TACE sessions was less than two. Therefore, the effect of additional TACE on HCC was expected to be sufficient in those studies. In addition, the STAB study (Sato et al., 2018) targeted patients with advanced disease, extrahepatic spread, and vascular invasion (Ogasawara et al., 2014; Arizumi et al., 2015; Ohki et al., 2015) examined the efficacy of sorafenib transfer in TACE-refractory patients with intermediate-stage HCC. To the best of our knowledge, the present study is the first to report the efficacy of sorafenib plus TACE in TACE-refractory patients with intermediate-stage HCC. No improvement in OS, which was the primary study endpoint, was observed in previous TACE combination trials (the OS results of the TACTICS trial have not been reported yet), but our study observed a significant improvement in OS in the combination treatment group. Previous studies of TACE combination therapy did not report an improvement in OS because even if TACE could be continued, protocol treatment was discontinued upon disease progression. It was suggested that the effect of sorafenib when combined with TACE could not be fully achieved because the sorafenib administration period in each study was short, ranging from 4.3 to 5.3 months. In the TACTICS study (Kudo et al., 2020), progressive disease was determined according to the time-to-untreatable (unTACEable) progression to assess when the benefit of TACE ceased, which is close to the actual clinical criteria. The duration of sorafenib treatment was 9.7 months in the TACTICS study, which was much longer than that in previous studies (Kudo et al., 2011; Lencioni et al., 2016; Meyer et al., 2017). As a result, that study showed a significant improvement in PFS, as the primary endpoint, in the TACE combination arm, and future OS analyses are expected to be performed. Our study targeted TACE-refractory patients, and additional TACE was performed as needed when new lesions appeared. The TACE discontinuation criteria in our study were relatively similar to those of the TACTICS trial. The duration of sorafenib administration was 11.6 months (median), which was longer compared with that in other studies (Kudo et al., 2011; Lencioni et al., 2016; Meyer et al., 2017), and our results suggested that this longer duration contributed to the longer OS. We evaluated differences in the timing of sorafenib introduction and resumption after TACE. In the Post-TACE study (Kudo et al., 2011), the interval from TACE to randomized assignment was 1.4–3.3 months; therefore, the long period before sorafenib administration may have been the cause of study failure (Kudo et al., 2011).

The cause of tumor progression after TACE is considered to be increased VEGF expression due to tumor ischemia, and administering sorafenib before and after TACE is expected to suppress the increase in VEGF levels. Furthermore, normalization of tumor vessels enables the uniform distribution of anticancer drugs and embolic substances within the tumor, and it is expected that the combination of sorafenib will enhance the effect of TACE (Sergio et al., 2008; Shim et al.; 2008 Wilhelm et al., 2008). In this study, sorafenib therapy was paused for 1 week after TACE; otherwise, treatment was continued without interruption. An improvement in the TTP, which is a beneficial antitumor effect of repeated TACE after patients become TACE refractory, and a longer interval between TACE rounds were expected; however, the TTP and antitumor effect of repeated TACE did not differ between the two treatment groups (Table 3). The interval between TACE rounds after the development of TACE refractoriness was significantly longer in the combination treatment group than in the TACE continuation group. Although additional TACE did not improve the treatment response rates (complete or partial response), the rate of stable disease tended to be higher in the combination treatment group than in the TACE continuation group (73% vs. 55%, respectively). It is possible that sorafenib slowed disease progression, leading to a longer interval between TACE rounds.

Major changes in HCC treatment have occurred recently. Since 2017, regorafenib, lenvatinib, ramucirumab, and atezolizumab plus bevacizumab as immunotherapy, have become available as treatments for advanced HCC. In particular, the combination of atezolizumab plus bevacizumab was the first treatment to demonstrate superiority to sorafenib in the IMbrave150 trial (Finn et al., 2020). However, a subgroup analysis of BCLC stage B patients did not show superiority of atezolizumab plus bevacizumab to sorafenib. Thus, the best treatment for TACE-refractory patients with BCLC stage B remains unclear. Considering these points and our results, we suggest that the combination of sorafenib and TACE for HCC patients who meet the definition of TACE refractoriness is still a sufficient treatment option in clinical practice.

In conclusion, combination treatment with sorafenib and TACE in TACE-refractory patients with intermediate-stage HCC resulted in prolongation of the time between TACE rounds, better maintenance of the hepatic reserve, improved transition rate to post-treatment, and significantly longer OS. The limitations of this study were its retrospective and non-randomized nature, relatively small sample size, and lack of standardization of the patient characteristics; thus, further research is needed.

*Abbreviations list*

TACE, transarterial chemoembolization; HCC, hepatocellular carcinoma; OS, overall survival; BCLC, Barcelona Clinic Liver Cancer; APASL, Asian Pacific Association for the Study of the Liver; AASLD, American Association for the Study of Liver Diseases; JSH, Japan Society of Hepatology; VEGF, vascular endothelial growth factor; PFS, progression-free survival; CT, computed tomography; MRI, magnetic resonance imaging; HR, hazard ratio; CI, confidence interval; PIVKA-II, protein induced by vitamin K absence-II; TTP, time to progression

## Author Contribution Statement

MK drafted and wrote the manuscript. KY contributed to the statistical analysis. HM, MI, HK, KM, SK, and MS participated in the study design and helped draft the manuscript. All authors contributed to the interpretation of the findings and read and approved the final manuscript.

## Data Availability

The datasets used and analyzed during the current study are available from the corresponding author upon reasonable request.

## Declarations

Ethics approval and consent to participate.

This study was conducted in accordance with the ethical principles of the Declaration of Helsinki and was approved by the Institutional Review Board of Kansai Medical University (IRB number: 2020050). Written informed consent was obtained from all patients and their families before performing the treatments.
